# Metabolomics analysis identifies differential metabolites and potential diagnostic biomarkers among pediatric sepsis subtypes

**DOI:** 10.1371/journal.pone.0351295

**Published:** 2026-06-11

**Authors:** Sisi Zhuang, Lili Zuo

**Affiliations:** The First Affiliated Hospital with Nanjing Medical University, Nanjing, Jiangsu, China; University of California Riverside, UNITED STATES OF AMERICA

## Abstract

**Background:**

Sepsis in children can be caused by a variety of pathogens, with bacteria and viruses being the most common. This study used metabolomics to identify differences in metabolic profiles and potential biomarkers among pathogens causing pediatric sepsis.

**Methods:**

Serum metabolomic profiles of pediatric bacterial and viral sepsis were obtained from the MetaboLights database (MTBLS563). Principal component analysis (PCA), partial least squares discriminant analysis (PLS-DA), and orthogonal PLS-DA were employed to explore metabolic distinctions. Differential expression metabolites (DEMs) were identified using the Wilcoxon rank-sum test and variable importance in projection (VIP) scores. Kyoto Encyclopedia of Genes and Genomes (KEGG) enrichment, receiver operating characteristic (ROC) analysis, Extreme Gradient Boosting (XGBoost) modeling, and Shapley Additive exPlanations (SHAP) analysis were conducted to determine diagnostic metabolites and evaluate model performance.

**Results:**

PCA and PLS-DA revealed distinct metabolic profiles among bacterial pediatric sepsis (PBID_PS), viral pediatric sepsis (VID_PS), and healthy controls. Fourteen differential metabolites were identified, primarily enriched in nitrogen metabolism, arginine biosynthesis, and the metabolism of alanine, aspartate, and glutamate. Among them, choline, glutamate, and glutamine exhibited strong discriminatory ability between PBID_PS and VID_PS. XGBoost and SHAP analyses confirmed these metabolites as key diagnostic indicators, achieving excellent predictive performance and revealing distinct metabolic reprogramming underlying different etiologies of pediatric sepsis.

**Conclusion:**

Metabolomic profiling revealed distinct metabolic signatures between bacterial and viral pediatric sepsis, with glutamate, glutamine, and choline serving as potential biomarkers.

## 1 Introduction

Pediatric sepsis (PS) is a life-threatening syndrome caused by a dysregulated host response to infection, characterized by systemic inflammation and multiple organ dysfunction [1,2]. It can be triggered by a wide range of pathogens, including bacteria, viruses [3,4]. In addition, host-related risk factors such as immunodeficiency, chronic underlying diseases, and invasive medical procedures may further increase susceptibility [5,6]. Globally, PS remains a major public health concern that seriously threatens the health and survival of children, with an estimated incidence of approximately 48 cases per 100,000 children and a mortality rate ranging from 10% to 25%, depending on disease severity and etiologic factors [7,8]. Although advances in intensive care, including early recognition, prompt antimicrobial therapy, and organ support, have improved patient outcomes to some extent, the overall prognosis of PS remains unsatisfactory [9,10]. This is largely due to substantial interindividual heterogeneity, complex pathological mechanisms, and the absence of reliable tools for risk stratification [11]. Current studies mainly focus on elucidating its immunopathogenesis, identifying prognostic biomarkers, and optimizing therapeutic strategies. However, establishing accurate and effective early diagnostic indicators, developing targeted therapeutic approaches, and constructing reliable prognostic evaluation systems remain critical scientific challenges that must be urgently addressed.

Metabolomics is a scientific discipline that investigates small-molecule metabolites and their dynamic changes within biological systems [12]. By systematically profiling metabolites, metabolomics can reveal metabolic characteristics under various physiological or pathological conditions, thereby providing crucial insights into disease mechanisms, early diagnosis, and biomarker discovery [13,14]. In recent years, metabolomic analyses have demonstrated that integrating clinical data with metabolic profiles enables the identification of characteristic metabolic alterations in patients with sepsis, elucidates individual variability and underlying pathological mechanisms, and facilitates early diagnosis, risk stratification, and clinical outcome prediction, thus supporting precision management of the disease [15]. Specifically, 532 differentially expressed metabolites (DEMs) have been identified in patients with sepsis, mainly affecting amino acid metabolism, phenylalanine metabolism, and tyrosine metabolism pathways [16]. Moreover, patients with multiple trauma complicated by sepsis exhibit significant alterations in amino acid, lipid, carbohydrate, and nucleotide metabolism [17]. Among these, nine potential biomarkers were identified, of which succinic semialdehyde, uracil, and uridine were validated for clinical diagnostic use, further underscoring the pivotal role of metabolomics in elucidating the mechanisms and improving the diagnosis of sepsis and its complex pathophysiological states.

**Fig 1:**
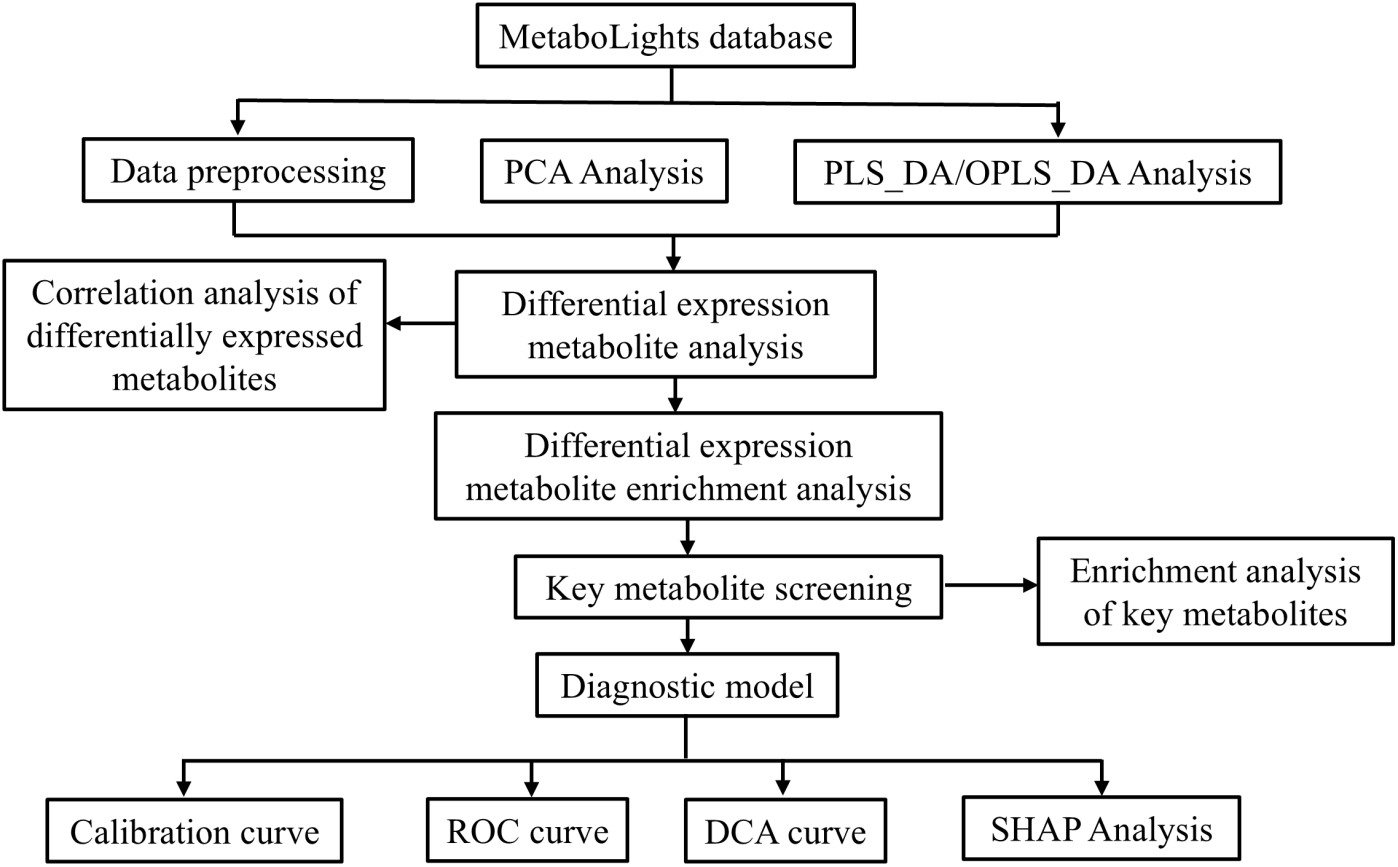
Flow chart.

Although PS is characterized by high incidence and clinical complexity, its pronounced etiological heterogeneity and challenges in early diagnosis continue to hinder the realization of precision treatment. In this study, serum metabolomics was employed to systematically characterize metabolic alterations between bacterial pediatric sepsis (PBID_PS) and viral pediatric sepsis (VID_PS) using principal component analysis (PCA), partial least squares discriminant analysis (PLS-DA), and orthogonal partial least squares discriminant analysis (OPLS-DA) (Fig 1). Through these comprehensive analyses, we aimed to identify key metabolic signatures and to provide insights into metabolic alterations associated with sepsis of different etiologies, thereby offering a theoretical basis and potential biomarker support for early diagnosis, etiological classification, and precision therapy in pediatric sepsis.

## 2. Materials and methods

### 2.1 Data preprocessing and normalization

Before multivariate statistical analysis, metabolomics data were preprocessed to improve comparability and reduce technical bias. Metabolites with more than 20% missing values were excluded. The remaining data were subjected to log_2_ transformation to reduce skewness and approximate normal distribution, followed by mean centering to eliminate the influence of magnitude differences among metabolites. Principal component analysis (PCA), partial least squares discriminant analysis (PLS-DA), and orthogonal PLS-DA (OPLS-DA) were performed using the R packages ropls and mixOmics.

### 2.2 Data collection and principal component analysis (PCA)

Publicly available serum metabolomic data from pediatric sepsis patients were retrieved from the MetaboLights database (https://www.ebi.ac.uk/metabolights/MTBLS563) on 15 June 2025. This dataset included serum metabolite profiles from 26 patients with primary bacterial infectious disease (PBID_PS, representing bacterial pediatric sepsis), 30 patients with viral infectious disease (VID_PS, representing viral pediatric sepsis), and 56 healthy controls (Control). It should be noted that this dataset does not include pediatric patients with fungal sepsis; therefore, fungal-related cases were not considered in the present analysis. PCA was performed using the R package ropls (v 1.24.0) to assess the overall metabolic distribution patterns among the PBID_PS, VID_PS, and Control groups. This unsupervised dimensionality reduction approach was applied to visualize clustering trends and evaluate the degree of separation among the three cohorts within a reduced feature space.

### 2.3 Metabolome discriminant analysis based on PLS-DA and OPLS-DA

Group-specific metabolic alterations were further explored using multivariate statistical approaches. Partial least squares discriminant analysis (PLS-DA) establishes a regression model between metabolite abundance and sample classification, thereby enabling prediction and discrimination of sample groups. The R package mixOmics (v 1.72.5) was used to perform PLS-DA on the entire dataset to evaluate global metabolic distribution patterns and identify discriminative metabolites. Scatter plots were generated to visualize the clustering tendency and separation among the groups. To evaluate model performance, seven-fold cross-validation was applied, and the predictive ability was assessed using the Q^2^ metric. Permutation testing (n = 1000) was further conducted to evaluate model robustness and statistical significance, generating pR^2^Y and pQ^2^ values. Additionally, orthogonal partial least squares discriminant analysis (OPLS-DA) was performed to further enhance model interpretability. This supervised method filters out orthogonal variations unrelated to class discrimination, thereby improving the analytical robustness and highlighting essential metabolic differences between groups.

### 2.4 Identification and functional annotation of differential expression metabolites (DEMs)

Differentially expressed metabolites (DEMs) among the three groups were determined by the Wilcoxon rank-sum test with thresholds set at p < 0.05 and variable importance in projection (VIP) > 1. Volcano plots were generated using all detected metabolites that passed quality control, rather than being restricted to DEMs, providing an intuitive overview of metabolite distribution across groups. To further investigate the relationships among these DEMs, Spearman’s correlation analysis was conducted using the R package corrplot (v 0.95) based on all samples. Metabolites showing an absolute correlation coefficient (|Cor|) > 0.3 and p < 0.05 were considered to exhibit significant associations. The threshold of |Cor| > 0.3 was selected based on conventional effect size interpretation, where |r| ≈ 0.3 represents a moderate correlation with potential biological relevance [18]. Considering the relatively limited sample size, this threshold was adopted to balance sensitivity and specificity, while the additional requirement of p < 0.05 was applied to reduce false-positive associations. The corresponding correlation network was then visualized with ggplot2 (v 3.5.1) to illustrate the interconnections among significant metabolites. Finally, to elucidate the potential biological pathways involved in these DEMs, Kyoto Encyclopedia of Genes and Genomes (KEGG) pathway enrichment analysis was performed using MetaboAnalyst 5.0 (https://www.metaboanalyst.ca/MetaboAnalyst/home.xhtml). Differential metabolites (DEMs) identified between groups were used as input for the analysis. The “Pathway Analysis” module was selected, and the organism was set to Homo sapiens. Pathway enrichment analysis was conducted using the hypergeometric test, and pathway topology analysis was performed using relative-betweenness centrality. Pathways with p < 0.05 were regarded as significantly enriched, and all such pathways were included in subsequent analyses.

### 2.5 Diagnostic evaluation and model building based on key metabolites

To evaluate the diagnostic and predictive potential of the identified DEMs, receiver operating characteristic (ROC) curve analysis was first performed based on dataset samples using the R package pROC (v 1.18.0). The area under the ROC curve (AUC) was calculated to quantify the diagnostic accuracy of each metabolite, where values between 0.7 and 1.0 were considered indicative of high diagnostic performance. Subsequently, an Extreme Gradient Boosting (XGBoost) model was constructed using the identified feature metabolites to further assess their collective discriminative ability. The risk of over-optimism during model construction was addressed by selecting features based on a combination of univariate significance (p < 0.05) and multivariate importance (VIP > 1) derived from permutation-validated OPLS-DA models. Additionally, SHAP analysis was integrated to ensure model interpretability and to prioritize metabolites with consistent contributions to the diagnostic output. ROC curves were plotted using the pROC package to assess the model’s ability to distinguish between bacterial and viral pediatric sepsis. Calibration curves were then generated to examine the agreement between predicted probabilities and observed outcomes, thereby validating the model’s reliability and accuracy. Furthermore, decision curve analysis (DCA) was conducted to assess the predictive model’s net clinical benefit across a range of threshold probabilities, providing insights into its potential clinical applicability. Finally, the relative importance of metabolites contributing to the diagnostic model was interpreted using Shapley Additive exPlanations (SHAP) analysis. SHAP values were computed to quantify the individual contribution of each metabolite to the model’s output, enabling the identification of key metabolites with the highest diagnostic relevance and potential clinical significance.

### 2.6 Statistical analysis

All statistical analyses were conducted using R software (v 4.2.2) unless otherwise specified. Continuous variables were expressed as mean ± standard deviation (SD) or median with interquartile range (IQR), depending on the data distribution assessed by the Shapiro-Wilk test. Comparisons between two groups were performed using the Wilcoxon rank-sum test, and multiple-group comparisons were conducted using the Kruskal-Wallis test, followed by post hoc pairwise analyses when appropriate.

## 3 Results

### 3.1 Metabolomic analysis reveals significant biological differences between bacterial pediatric sepsis (PBID_PS) and viral pediatric sepsis (VID_PS)

**Fig 2:**
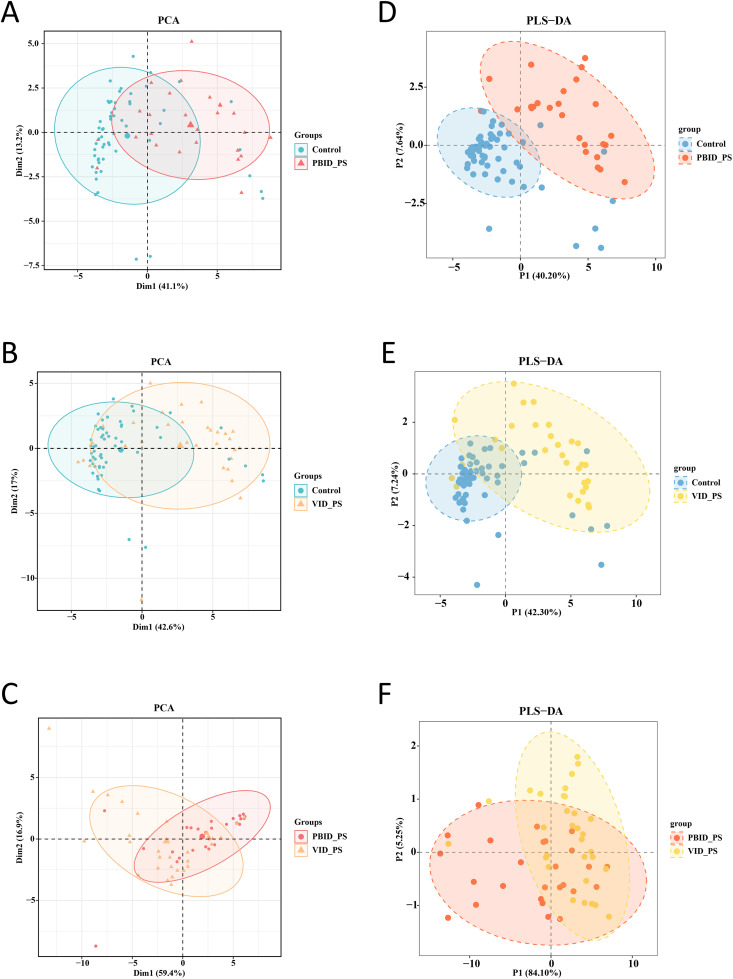
Metabolomic analysis reveals significant biological differences between bacterial pediatric sepsis (PBID_PS) and viral pediatric sepsis (VID_PS). **(A-C)** Principal component analysis of bacterial pediatric sepsis (PBID_PS) vs. control **(A)**, viral pediatric sepsis (VID_PS) vs. control **(B)**, and PBID_PS vs. VID_PS **(C)**. PBID_PS represents primary bacterial infectious disease corresponding to bacterial pediatric sepsis, and VID_PS represents viral infectious disease corresponding to viral pediatric sepsis. The pink, orange, and blue colors indicate PBID_PS, VID_PS, and control groups, respectively. **(D-F)** Partial least squares discriminant analysis (PLS-DA) score plots for PBID_PS vs. control **(D)**, VID_PS vs. control **(E)**, and PBID_PS vs. VID_PS **(F)**. Each point represents an individual sample, and the relative positions reflect the similarity or dissimilarity of metabolic profiles among samples.

A total of 35 metabolites were identified based on LC-MS/MS analysis (S1–S3 Tables). Given the distinct clinical manifestations of bacterial and viral sepsis, an initial global assessment of metabolic variability was conducted to explore whether the three groups exhibit divergent metabolic patterns. PCA revealed a clear separation between the sepsis groups and the control group, whereas the separation between bacterial pediatric sepsis (PBID_PS) and viral pediatric sepsis (VID_PS) was less distinct, with partial overlap observed (Fig 2A–2C). Partial overlap among groups was observed in the PCA score plot, likely reflecting shared metabolic features and intrinsic heterogeneity among pediatric sepsis patients. Notably, PBID_PS and VID_PS exhibited distinct distributions along the principal components, suggesting fundamentally different underlying mechanisms. Consistently, the PLS-DA model based on metabolomic data clearly distinguished bacterial sepsis (PBID_PS), viral sepsis (VID_PS), and controls (Fig 2D–2F). These findings demonstrate that unique metabolic signatures characterize pediatric sepsis of different etiologies.

### 3.2 OPLS-DA revealed significant metabolic differences between childhood bacterial pediatric sepsis (PBID_PS) and viral pediatric sepsis (VID_PS)

**Fig 3:**
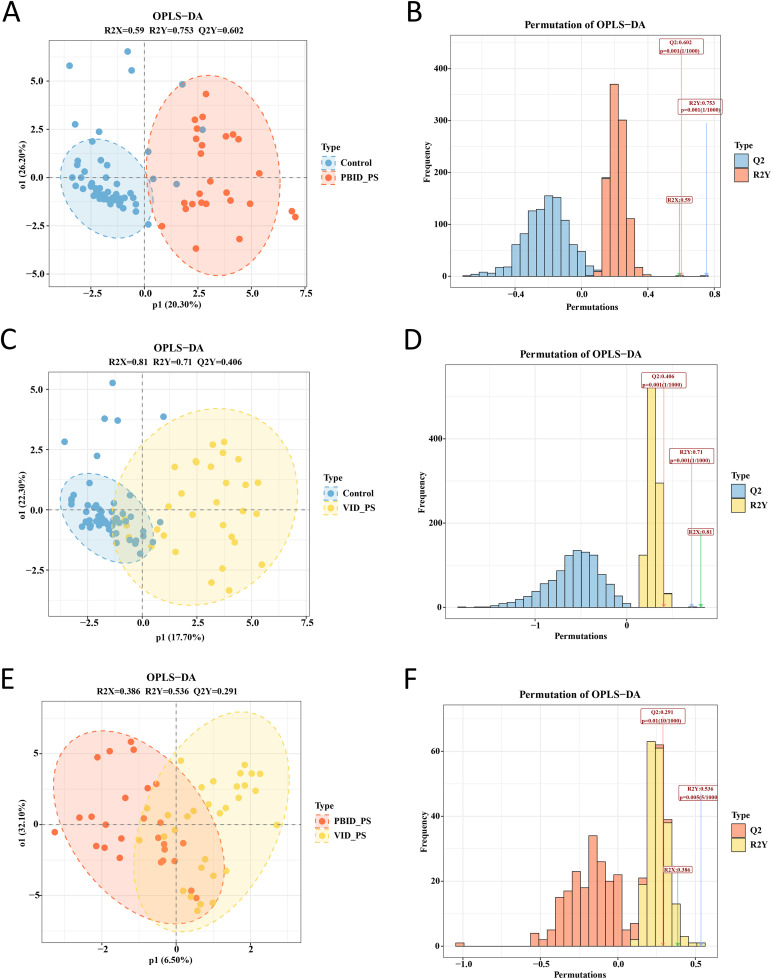
OPLS-DA revealed significant metabolic differences between childhood bacterial pediatric sepsis (PBID_PS) and viral pediatric sepsis (VID_PS). **(A)** OPLS-DA score plot for bacterial pediatric sepsis (PBID_PS) vs. control. **(B)** Permutation test plot for PBID_PS vs. control. **(C)** OPLS-DA score plot for viral pediatric sepsis (VID_PS) vs. control. **(D)** Permutation test plot for VID_PS vs. control. **(E)** OPLS-DA score plot for PBID_PS vs. VID_PS. **(F)** Permutation test plot for PBID_PS vs. VID_PS. PBID_PS represents primary bacterial infectious disease corresponding to bacterial pediatric sepsis, and VID_PS represents viral infectious disease corresponding to viral pediatric sepsis.

While PCA provided an overview of global metabolic variation, this method does not optimize class discrimination. Therefore, OPLS-DA was further employed to enhance group separation and identify metabolic features specifically contributing to the differences between PBID_PS and VID_PS. OPLS-DA was applied to distinguish PBID_PS, VID_PS, and control groups based on metabolomic profiles. In the PBID_PS versus control comparison, permutation tests yielded pR^2^Y = 0.001 and pQ^2^ = 0.001, indicating high model accuracy (S1A Fig). The model exhibited a Q^2^Y value of 0.602, suggesting good predictive performance and no evidence of overfitting (Fig 3A–3B). Similarly, in the VID_PS versus control comparison, permutation tests (pR^2^Y = 0.001, pQ^2^ = 0.001) confirmed model robustness, with Q^2^Y = 0.406 (Fig 3C–3D, S1C Fig). The model remained stable and reliable for identifying differential metabolites. For the PBID_PS versus VID_PS comparison, permutation tests showed pR^2^Y = 0.005 and pQ^2^ = 0.01, confirming adequate model validity (Fig S1E). The lower Q^2^Y value (0.291) reflected the intrinsic similarity between the two sepsis subgroups, yet the model retained predictive capacity (R^2^Y = 0.536, Q^2^ > 0, p = 0.01), demonstrating that the separation was not random (Figs 3E–3F). The top 30 metabolites were selected based on VIP values for visualization (S1B, S1D, and S1F Figs).

### 3.3 Metabolic differences between bacterial pediatric sepsis (PBID_PS) and viral pediatric sepsis (VID_PS)

**Fig 4:**
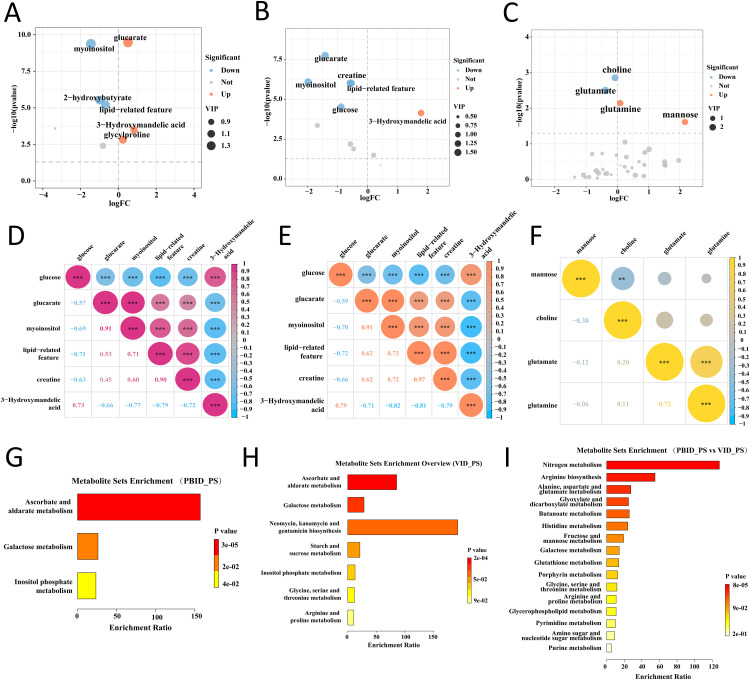
Metabolic differences between bacterial pediatric sepsis (PBID_PS) and viral pediatric sepsis (VID_PS). **(A)** Volcano plot of the PBID_PS vs. the Control groups. **(B)** Volcano plot of the VID_PS vs. the Control groups. **(C)** Volcano plot of the PBID_PS vs. VID_PS groups. **(D)** Correlation analysis between DEMs of the PBID_PS vs. the Control groups. **(E)** Correlation analysis between DEMs of the VID_PS vs. the Control groups. **(F)** Correlation analysis between DEMs of the PBID_PS vs. VID_PS groups. Pink, orange, and yellow colors indicate positive correlations in the three graphs, respectively, while blue indicates negative correlations. **(G)** KEGG metabolic pathway analysis of DEMs of the PBID_PS vs. the Control groups. **(H)** KEGG metabolic pathway analysis of DEMs of the VID_PS vs. the Control groups. **(I)** KEGG metabolic pathway analysis of DEMs of the PBID_PS vs. VID_PS groups.

After establishing the overall metabolic differences across groups, we next focused on identifying specific metabolites that distinguish PBID_PS from VID_PS. Volcano plot analysis identified six differential metabolites (DEMs) in both PBID_PS and VID_PS compared with controls, yet their expression patterns differed markedly (Fig 4A–4C). Direct comparison between PBID_PS and VID_PS revealed four significantly altered metabolites. Correlation analyses of these DEMs (Fig 4D–4F) indicated extensive inter-metabolite associations within each group. In PBID_PS, strong positive correlations were observed between myo-inositol and glucarate, while 3-hydroxymandelic acid showed a significant negative correlation with the lipid-related feature. In VID_PS, creatine displayed a strong positive correlation with the lipid-related feature and a negative correlation with 3-hydroxymandelic acid. Within the PBID_PS versus VID_PS comparison, glutamine was positively correlated with glutamate, whereas choline was inversely correlated with mannose. KEGG pathway enrichment analysis further revealed distinct metabolic pathway alterations across groups (Fig 4G–4I). The six DEMs in PBID_PS were significantly enriched in ascorbate and aldarate metabolism, galactose metabolism, and inositol phosphate metabolism. In VID_PS, the DEMs were mainly enriched in ascorbate and aldarate metabolism, galactose metabolism, and aminoglycoside biosynthesis pathways. In contrast, the four DEMs distinguishing PBID_PS from VID_PS were predominantly involved in nitrogen metabolism, arginine biosynthesis, and the metabolism of alanine, aspartate, and glutamate, highlighting the divergent metabolic reprogramming between PBID_PS and VID_PS.

### 3.4 ROC curve analysis identified key DEMs in pediatric sepsis subtypes

**Fig 5:**
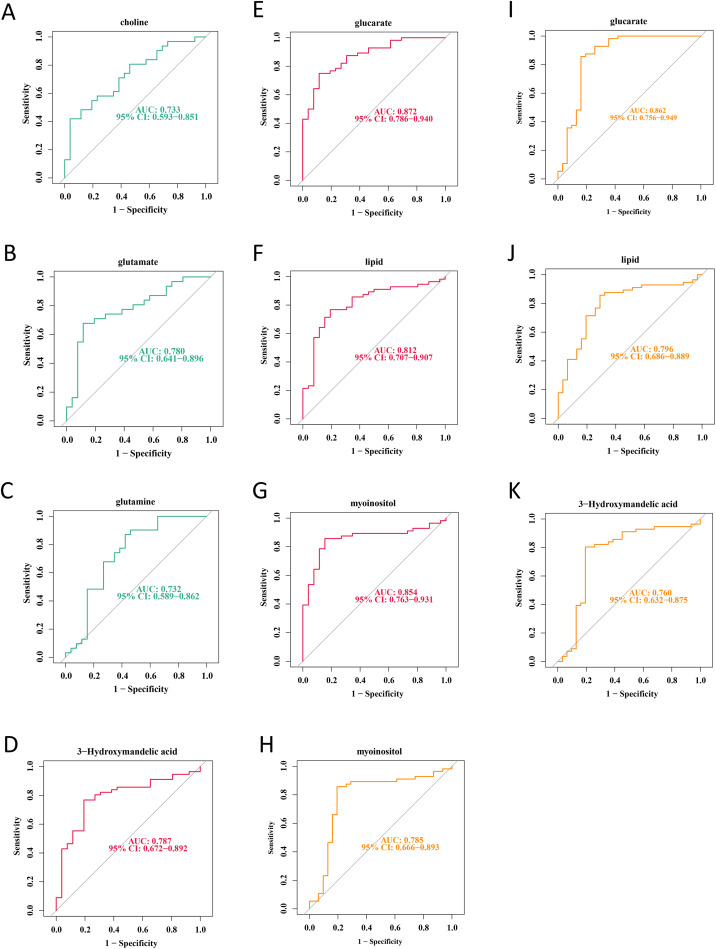
ROC curve analysis identified key DEMs in pediatric sepsis subtypes. **(A-C)** ROC curves of DEMs for PBID_PS vs. VID_PS. **(D)** KEGG metabolic pathway analysis of key DEMs. **(D-G)** ROC curves of DEMs for PBID_PS vs. Control. **(H-K)** ROC curves of DEMs for VID_PS vs. Control. The vertical axis (Sensitivity) represents sensitivity, i.e., the true positive rate; the horizontal axis (Specificity) represents specificity, i.e., the false positive rate; the diagonal line represents the null curve, indicating random guessing.

Given the metabolic distinctions identified above, assessing their diagnostic performance became a critical next step. ROC curve analysis was performed to evaluate the diagnostic potential of differential metabolites. In the comparison between PBID_PS and VID_PS, choline, glutamate, and glutamine exhibited AUC values exceeding 0.7, suggesting their high discriminative power (Fig 5A–5C). However, mannose has an AUC value of <0.7, so choline, glutamate, and glutamine were identified as the key metabolites (S2A Fig). In the PBID_PS versus control comparison, six metabolic features, including glucarate, myo-inositol, 2-hydroxybutyrate, a lipid-related feature, 3-hydroxymandelic acid, and glycylproline, showed AUC values above 0.7, identifying them as key biomarkers capable of distinguishing PBID_PS from healthy controls (Fig 5D–5G, S2B–S2C Figs). Similarly, in the VID_PS versus control analysis, six metabolites, including glucarate, myo-inositol, choline, lipid, glucose, and 3-hydroxymandelic acid, demonstrated AUC values greater than 0.7, indicating strong predictive performance for VID_PS identification (Fig 5H–5K, S2D–S2E Figs).

### 3.5 Identification of key metabolites in pediatric sepsis subtypes by XGBoost modeling and SHAP analysis

**Fig 6:**
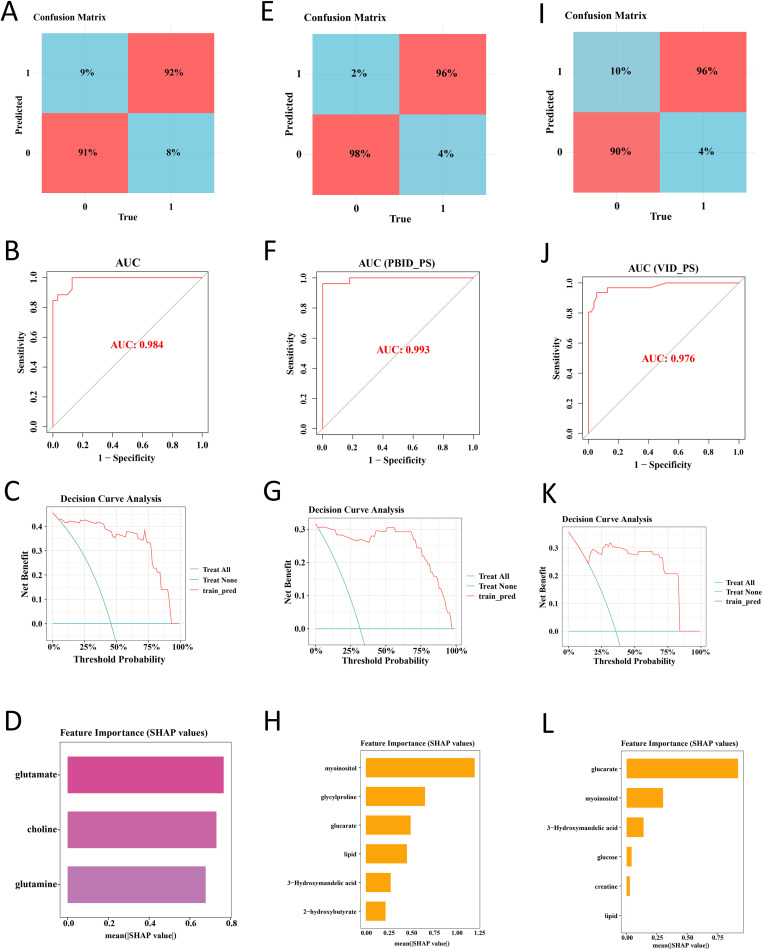
Identification of key metabolites in pediatric sepsis subtypes by XGBoost modeling and SHAP analysis. **(A-D) (A)** Confusion matrix, **(B)** ROC curve, **(C)** DCA decision curve, and **(D)** SHAP variable importance bar chart for the PBID_PS and VID_PS datasets. **(E-H) (E)** Confusion matrix, **(F)** ROC curve, **(G)** DCA decision curve, and **(H)** SHAP variable importance bar chart for the PBID_PS and Control datasets. **(I-L) (I)** Confusion matrix, **(J)** ROC curve, **(K)** DCA decision curve, and **(L)** SHAP variable importance bar chart for the VID_PS and Control datasets.

A single ROC curve value may not fully reflect the diagnostic value of these metabolites. Therefore, XGBoost multivariate modeling technology was further used to construct models that can distinguish different subtypes, and the diagnostic performance of these models was systematically evaluated. In the PBID_PS vs. VID_PS model, the AUC reached 0.984, indicating strong discriminative power (Fig 6A–6B). The calibration curve exhibited a high degree of overlap between predicted and actual probabilities (S3A Fig). DCA further demonstrated favorable clinical utility (Fig 6C). SHAP analysis revealed that glutamate ranked first in global importance, exerting the greatest influence on model prediction (Fig 6D, S3B Fig). Collectively, choline, glutamate, and glutamine were identified as key discriminative metabolites for PBID_PS and VID_PS. Similarly, the PBID_PS vs. Control model yielded an AUC of 0.993, reflecting excellent diagnostic capability (Fig 6E–6F, S3C Fig). The DCA results confirmed its high predictive efficiency (Fig 6G). Among all variables, myoinositol showed the highest global importance and the most dispersed SHAP value distribution, indicating its dominant role in prediction (Fig 6H, S3D Fig). For the VID_PS vs. Control model, the AUC value was 0.976, suggesting robust discriminative performance (Fig 6I–6J, S3E Fig). The DCA curve supported its predictive validity (Fig 6K). Glucarate was ranked first in global importance and showed the widest SHAP distribution, highlighting its major contribution to the model (Fig 6L, S3F Fig). Overall, these key metabolites demonstrated strong diagnostic potential in distinguishing different sepsis etiologies.

## 4 Discussion

Pediatric sepsis is a systemic inflammatory response syndrome triggered by infection, characterized by rapid disease progression and high mortality, making early diagnosis and etiological determination critically important [19,20]. Metabolomics, through high-throughput detection of small-molecule metabolites in blood or tissues, provides a comprehensive reflection of metabolic alterations within the body and serves as a powerful approach for elucidating disease mechanisms and identifying potential biomarkers [21,22]. Therefore, metabolomics holds great promise for distinguishing etiologies and improving diagnostic accuracy in pediatric sepsis. In the present study, serum metabolomic profiling revealed distinct metabolic characteristics between PBID_PS and VID_PS. PCA, PLS-DA, and OPLS-DA all demonstrated clear separation between groups, indicating fundamentally different metabolic patterns. Further analysis of differential metabolites and KEGG pathway enrichment revealed metabolic reprogramming involving nitrogen metabolism, arginine biosynthesis, and glutamate-related pathways in both sepsis subtypes. Moreover, ROC analysis and XGBoost modeling confirmed that metabolites such as choline, glutamate, and glutamine possessed robust diagnostic value in distinguishing sepsis subtypes, highlighting their potential as biomarkers for etiological classification and early diagnosis.

This study provides a secondary analysis of the MTBLS563 dataset originally generated by Grauslys et al. [23]. While the original study focused on OPLS-DA and PCA, we expanded the analytical framework by incorporating PLS-DA, Spearman correlation, and XGBoost with SHAP interpretability. Our findings regarding key metabolites like glucarate and myo-inositol are consistent with the original results. However, our integration of machine learning offers a more granular assessment of diagnostic potential, extending the biological insights provided by the initial study. Building upon the global metabolic distinctions identified in our analysis, PCA and PLS-DA further demonstrated clear separations among PBID_PS, VID_PS, and healthy controls, suggesting that bacterial and viral infections induce fundamentally distinct patterns of metabolic reprogramming. This finding is consistent with previous studies reporting that different pathogens can alter host metabolic states through pathways related to inflammation, energy metabolism, and immune regulation [24,25]. Supporting evidence from Li et al. showed that serum metabolomic profiles of pediatric sepsis patients differed significantly from those of healthy controls and could effectively discriminate among various infectious etiologies [26]. Similarly, Bian et al. identified multiple potential metabolic biomarkers in neonatal sepsis, providing new insights for clinical diagnosis [27]. Collectively, these studies corroborate the promising value of metabolomics in investigating pediatric sepsis. Furthermore, OPLS-DA validation confirmed the robustness and predictive performance of our model without signs of overfitting, indicating that differential metabolites can reliably distinguish between sepsis subtypes.

Notably, the relatively low Q^2^Y values observed between bacterial pediatric sepsis (PBID_PS) and viral pediatric sepsis (VID_PS) indicate substantial similarity in their global metabolic profiles. This value specifically suggests a high degree of metabolic overlap between the two sepsis types, which is biologically plausible as both bacterial and viral infections trigger common host response pathways and systemic inflammatory cascades. Although this overlap reflects shared pathophysiological features, the significant permutation test results (p = 0.01) confirm that the model remains valid and can identify subtle yet critical etiological differences. It is also important to consider that the metabolic signatures of pediatric sepsis may be influenced by the specific type of infecting pathogen. Previous studies have demonstrated that Gram-negative bacteria such as E. coli and Gram-positive bacteria such as S. aureus can induce distinct metabolic profiles due to their unique pathogen-associated molecular patterns. Gram-negative infections often trigger a more rapid shift toward glycolytic pathways and different alterations in the tricarboxylic acid cycle compared to Gram-positive infections [28]. Similarly, different viral etiologies may also contribute to the observed metabolic heterogeneity.

Because the current dataset (MTBLS563) lacks detailed information regarding specific bacterial and viral strains for each patient, the identified signatures represent a generalized host response to these broad categories of infection. This inherent heterogeneity likely contributes to the partial overlap observed in the multivariate models and warrants further investigation using larger cohorts with precise microbiological characterization. The partial overlap observed between bacterial and viral pediatric sepsis in multivariate analyses may reflect shared systemic inflammatory responses. However, due to the lack of detailed clinical metadata, including disease severity scores, the underlying drivers of this overlap could not be further explored and warrant investigation in future studies. These findings indicate that serum metabolomics not only uncovers characteristic metabolic alterations associated with distinct etiologies of pediatric sepsis but also provides a valuable foundation for developing early diagnostic biomarkers and etiological classification tools.

Serum metabolomic analysis revealed significant differences in metabolites between both PBID_PS and VID_PS compared with healthy controls, and intrinsic distinctions in metabolic patterns and related pathways between the two sepsis subtypes. Specifically, differential metabolites in PBID_PS were mainly enriched in ascorbate and aldarate metabolism, galactose metabolism, and inositol phosphate metabolism, whereas VID_PS was more closely associated with aminoglycoside biosynthesis. The enrichment of the aminoglycoside biosynthesis pathway in viral infections likely represents host metabolic reprogramming of amino sugar and nucleotide sugar precursors. While this pathway is traditionally linked to microbial antibiotic production, its identification in human serum indicates significant shifts in the hexosamine biosynthetic pathway. Viral replication relies heavily on host glycosylation machinery for the synthesis and modification of viral envelope proteins [29]. Additionally, amino sugars serve as essential building blocks for inflammatory signaling and the modulation of host immune responses through O-GlcNAcylation during viral challenges. These distinct metabolic requirements for viral protein assembly and host defense mechanisms may explain why this pathway is more prominently altered in viral pediatric sepsis compared to bacterial cases. Further comparison indicated that the differential metabolites distinguishing the two subtypes were primarily involved in nitrogen metabolism, arginine biosynthesis, and glutamate-related pathways. Consistent with our findings, neonatal sepsis complicated by meningitis has been reported to exhibit significant decreases in serum and cerebrospinal fluid levels of arginine and its derivatives, along with marked alterations in creatine and related metabolites, suggesting that arginine metabolism and nitric oxide synthesis pathways are disrupted [30]. In addition, key genes identified in pediatric sepsis (UPP1, S100A9, KIF1B, S100A12, and SLC26A8) were found to be significantly enriched in galactose, fructose-mannose, and starch-sucrose metabolism pathways based on Gene Set Enrichment Analysis (GSEA) analysis, further indicating that galactose metabolism is profoundly altered in pediatric sepsis [31]. Collectively, these findings demonstrate that pediatric sepsis of different etiologies exhibits distinct metabolic reprogramming patterns, and the identified key metabolites may serve as potential subtype-specific biomarkers, providing important insights into the underlying pathophysiological mechanisms and aiding clinical diagnosis.

Given the distinct metabolic alterations observed between PBID_PS and VID_PS, the diagnostic performance of these differential metabolites was further evaluated through ROC curve analysis. Choline, glutamate, and glutamine exhibited strong discriminatory ability in differentiating PBID_PS from VID_PS. At the same time, metabolites such as myo-inositol, glucarate, 2-hydroxybutyric acid, several lipid species, and 3-hydroxymandelic acid demonstrated significant capacity to distinguish sepsis subtypes from healthy controls. Glutamate and choline serve as critical indicators of the systemic inflammatory response in pediatric sepsis. Glutamate, a major excitatory amino acid neurotransmitter, plays a central role in nitrogen metabolism and the glutamate-glutamine cycle [32,33]. Glutamate levels fluctuate during systemic inflammation as this metabolite supports the increased metabolic demands of activated immune cells. This amino acid also contributes to the synthesis of glutathione, which is the primary antioxidant required to neutralize reactive oxygen species during sepsis. In pediatric sepsis-associated encephalopathy (SAE), exogenous glutathione (GSH) has been shown to alleviate lipid peroxidation, suppress inflammatory cytokines, and improve neurofunctional markers by modulating glutamate-related metabolism, thereby exerting neuroprotective effects [34]. Choline, an essential precursor for membrane phospholipid synthesis and acetylcholine production, may influence immune regulation and inflammatory responses during sepsis, reflecting systemic metabolic reprogramming [35,36]. Specifically, choline acts as a precursor for acetylcholine, the primary neurotransmitter of the cholinergic anti-inflammatory pathway. This pathway modulates the host inflammatory response by inhibiting the release of pro-inflammatory cytokines through the nicotinic acetylcholine receptor. Consequently, alterations in choline levels reflect both the structural damage to cell membranes caused by inflammatory mediators and the host’s attempt to regulate systemic inflammation through neuro-immune interactions. Moreover, abnormal levels of choline and its related metabolites have been observed in plasma and urine samples of patients with sepsis-associated acute kidney injury (SA-AKI), accompanied by altered expression of choline-metabolizing enzymes. Experimental studies have demonstrated that choline supplementation improves renal function in septic mice, suggesting a potential protective role of choline in pediatric sepsis [37]. Furthermore, SHAP analysis identified glutamate and myo-inositol as the most influential variables in the prediction model, collectively supporting that these key metabolites can effectively discriminate between sepsis etiologies and subtypes, thus offering high diagnostic and prognostic value.

From a clinical perspective, early differentiation between bacterial and viral sepsis remains a major challenge and is closely linked to prognosis and therapeutic decision-making. In this context, the metabolite signatures identified in the present study, particularly choline, glutamate, and glutamine, exhibited strong discriminatory performance and may serve as candidate biomarkers for rapid etiological classification. This distinction is clinically critical because inappropriate or delayed treatment can lead to adverse outcomes. For example, unnecessary antibiotic use in viral infections or delayed antimicrobial therapy in bacterial sepsis may worsen disease progression. Therefore, integrating metabolomics-based biomarkers with machine learning approaches may enhance diagnostic precision, facilitate timely and targeted interventions, and ultimately improve clinical outcomes in pediatric sepsis. Other differential metabolites in the volcano plots also reflect the pathophysiological state of pediatric sepsis. For instance, 3-hydroxymandelic acid indicates sympathetic nervous system activation and oxidative stress, while glycylproline reflects accelerated protein catabolism and tissue remodeling. These metabolic markers offer complementary value to the current clinical standard of care. Traditionally, clinicians rely on C-reactive protein (CRP), procalcitonin (PCT), and lactate to identify systemic infection and monitor tissue perfusion. However, these conventional indicators frequently demonstrate limited specificity in differentiating bacterial from viral etiologies. The integration of biomarkers like 3-hydroxymandelic acid and glycylproline could enhance diagnostic precision by capturing specific metabolic disturbances that are often missed by standard inflammatory proteins. This approach aligns with the growing need for precision medicine to facilitate targeted therapeutic interventions in pediatric critical care.

Overall, this study, based on serum metabolomic profiling, revealed pronounced metabolic reprogramming in both PBID_PS and VID_PS, with fundamentally distinct metabolic patterns and pathways between the two pediatric sepsis subtypes. Specifically, PBID_PS was characterized by alterations in ascorbate and aldarate metabolism, galactose metabolism, and inositol phosphate metabolism, whereas VID_PS primarily involved aminoglycoside biosynthesis. Further pathway analysis indicated that nitrogen metabolism, arginine biosynthesis, and glutamate-related pathways were notably distinct between the two groups. ROC curve and XGBoost model validations further confirmed that key metabolites such as choline, glutamate, and glutamine possess strong diagnostic and predictive potential, suggesting their utility as biomarkers for subtype differentiation.

However, several limitations should be acknowledged. First, the relatively small sample size and the absence of an independent external validation cohort may restrict the generalizability of these findings. Although permutation tests and SHAP analysis were employed to mitigate the risk of over-optimism, future confirmation in larger pediatric sepsis populations remains essential. Second, this study relied on computational and metabolomic analyses without in vivo or in vitro experimental validation to further elucidate the biological roles of the identified metabolites. Third, the dataset was limited to bacterial and viral infections, thereby excluding other pathogen types such as fungal sepsis and potentially overlooking broader metabolic heterogeneity. Fourth, the limited availability of clinical metadata and severity indicators in the public dataset precluded a comprehensive baseline characterization and correlation analysis with disease progression. Fifth, KEGG pathway enrichment was performed using a relatively small number of differential metabolites and a global reference set, which may introduce some bias in the enrichment results.

## 5 Conclusions

This study demonstrates that PBID_PS and VID_PS origins exhibit distinct metabolic profiles, reflecting divergent pathophysiological mechanisms. Multivariate analyses, including PCA, PLS-DA, and OPLS-DA, revealed clear separations among PBID_PS, VID_PS, and healthy controls. In contrast, correlation and pathway enrichment analyses highlighted differential engagement of ascorbate and aldarate metabolism, galactose metabolism, inositol phosphate metabolism, nitrogen metabolism, arginine biosynthesis, and glutamate-related pathways. ROC and XGBoost models identified key discriminative metabolites, notably choline, glutamate, glutamine, myo-inositol, and glucarate, with high predictive performance for distinguishing sepsis subtypes. SHAP analysis further emphasized the central roles of glutamate and myo-inositol in model predictions, underscoring their potential as biomarkers. Collectively, these findings reveal etiology-specific metabolic reprogramming in pediatric sepsis, suggesting that targeted metabolite profiling could enhance early diagnosis, subtype differentiation, and inform precision therapeutic strategies for children with sepsis.

## Supporting information

S1 Fig (A) PBID permutation test plot for the OPLS-DA analysis of PBID_PS vs. Control. (B) VIP lollipop plot of the top 30 metabolites for the OPLS-DA analysis of PBID_PS vs. Control. (C) PBID permutation test plot for the OPLS-DA analysis of VID_PS vs. Control. (D) VIP lollipop plot of the top 30 metabolites for the OPLS-DA analysis of VID_PS vs. Control. (E) PBID permutation test plot for the OPLS-DA analysis of PBID_PS vs. VID_PS. (F) VIP lollipop plot of the top 30 metabolites for the OPLS-DA analysis of PBID_PS vs. VID_PS.
(TIF)

S2 Fig (A) ROC curve for mannose in PBID_PS vs. VID_PS. (B-C) ROC curves for DEMs (2-hydroxybutyrate and glycylproline) in PBID_PS vs. Control. (D-E) ROC curves for DEMs (creatine and glucose) in VID_PS vs. Control.
(TIF)

S3 Fig (A-B) (A) Calibration curves and (B) SHAP bee swarm plots for the PBID_PS and VID_PS datasets. (C-D) (C) Calibration curves and (D) SHAP bee swarm plots for the PBID_PS and Control datasets. (E-F) (E) Calibration curves and (F) SHAP bee swarm plots for the VID_PS and Control datasets.
(TIF)

S1 Table The list of PBID_PS_DEM. (XLSX)

S2 Table The list of VID_PS_DEM. (XLSX)

S3 Table The list of PBID_PS vs VID_PS_DEM. (XLSX)

## Abbreviations

PS: Pediatric Sepsis
PBID_PS: Pediatric Bacterial Infectious Disease Sepsis
VID_PS: Viral Infectious Disease Pediatric Sepsis
PCA: Principal Component Analysis
PLS-DA: Partial Least Squares Discriminant Analysis
OPLS-DA: Orthogonal Partial Least Squares Discriminant Analysis
DEMs: Differential Expression Metabolites
VIP: Variable Importance in Projection
KEGG: Kyoto Encyclopedia of Genes and Genomes
ROC: Receiver Operating Characteristic
AUC: Area Under the ROC Curve
SHAP: Shapley Additive exPlanations
DCA: Decision Curve Analysis
