# Supplementary figures and images for: Metabolomics analysis identifies differential metabolites and potential diagnostic biomarkers among pediatric sepsis subtypes
Source: PLoS One. 2026 Jun 11;21(6):e0351295. doi: 10.1371/journal.pone.0351295 (PMC13257987; doi:10.1371/journal.pone.0351295)

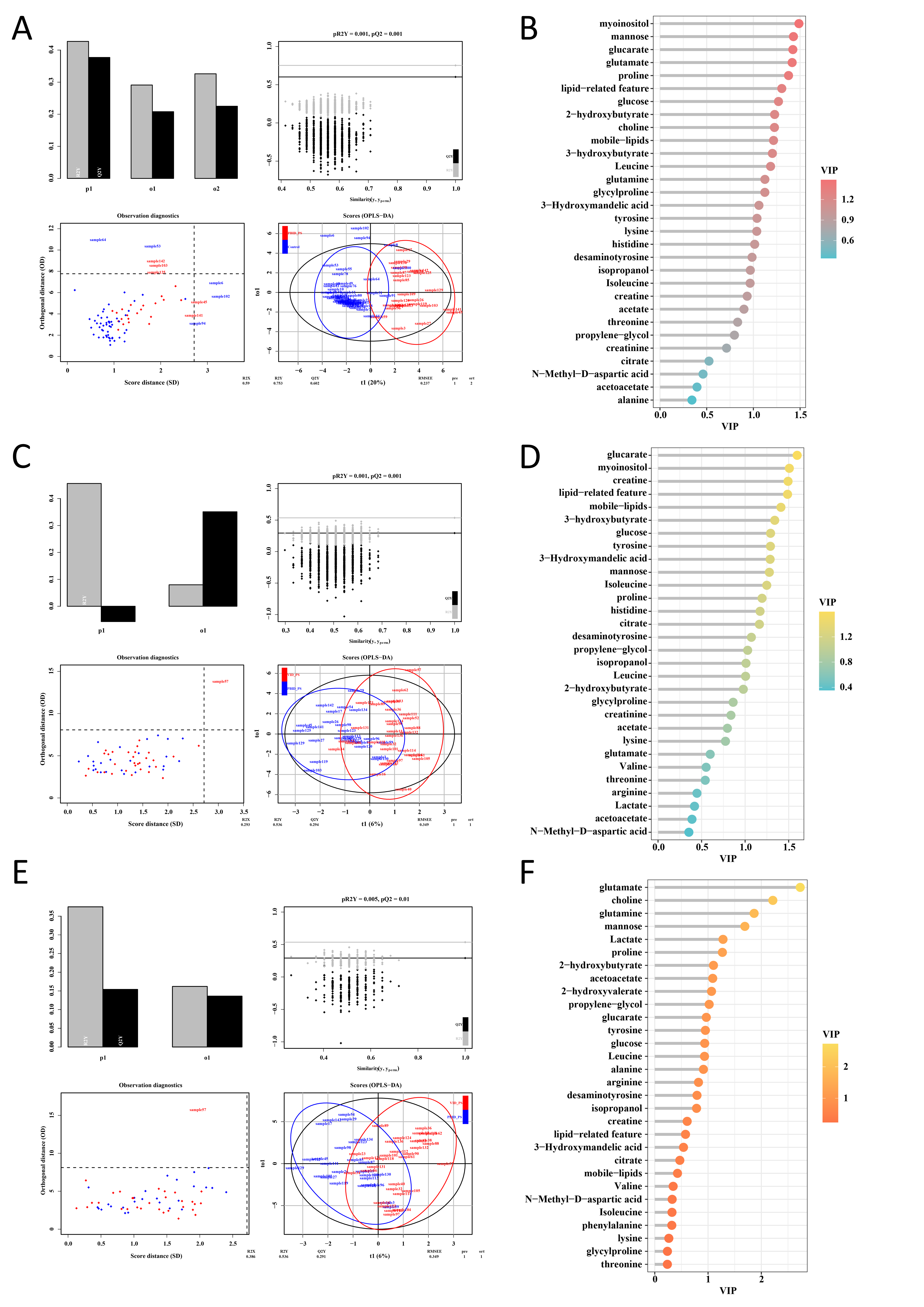

Supplement: S1 Fig — (B) VIP lollipop plot of the top 30 metabolites for the OPLS-DA analysis of PBID_PS vs. Control. (C) PBID permutation test plot for the OPLS-DA analysis of VID_PS vs. Control. (D) VIP lollipop plot of the top 30 metabolites for the OPLS-DA analysis of VID_PS vs. Control. (E) PBID permutation test plot for the OPLS-DA analysis of PBID_PS vs. VID_PS. (F) VIP lollipop plot of the top 30 metabolites for the OPLS-DA analysis of PBID_PS vs. VID_PS. (TIF) [file pone.0351295.s001.TIF]

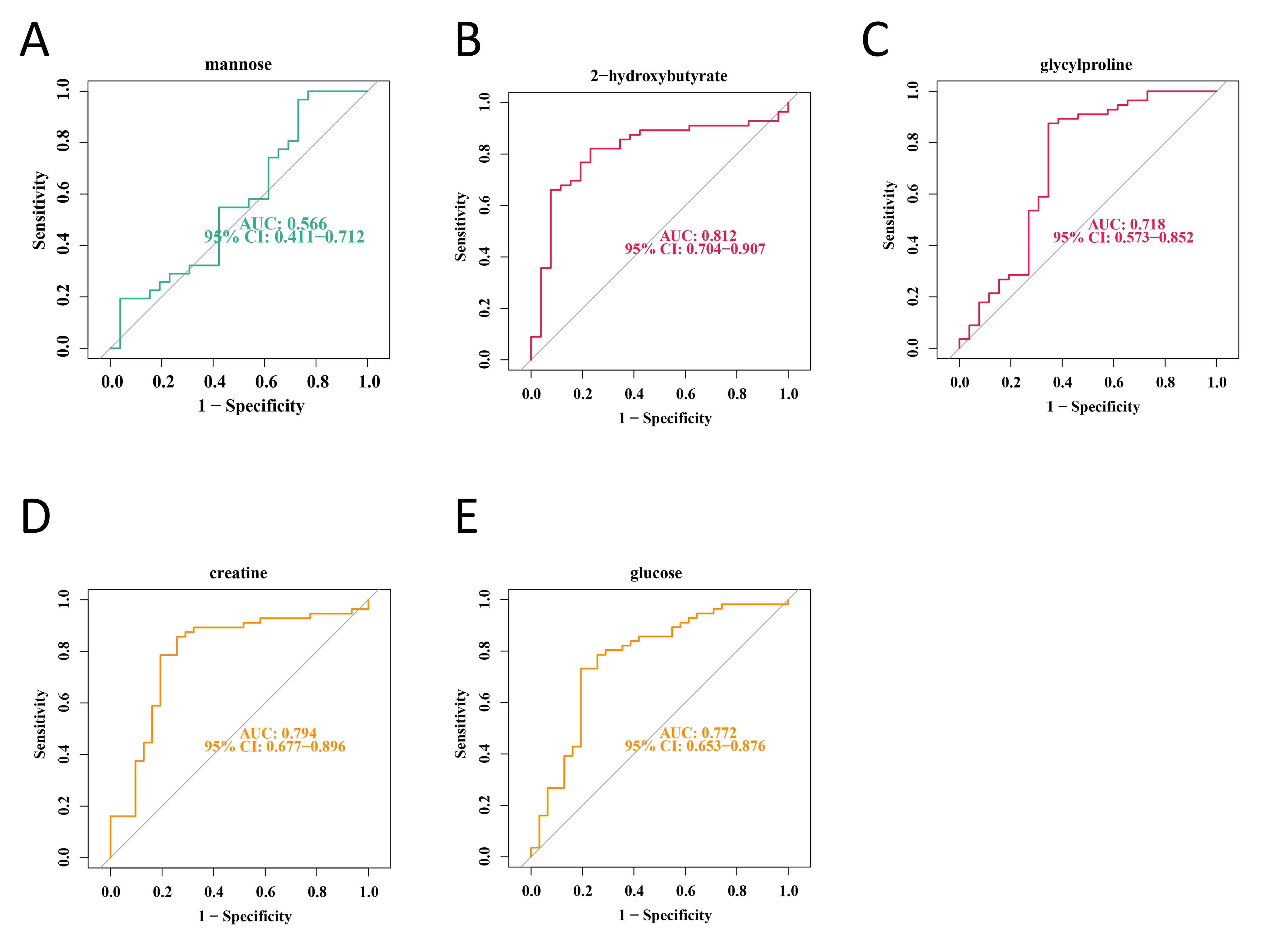

Supplement: S2 Fig — (D-E) ROC curves for DEMs (creatine and glucose) in VID_PS vs. Control. (TIF) [file pone.0351295.s002.TIF]

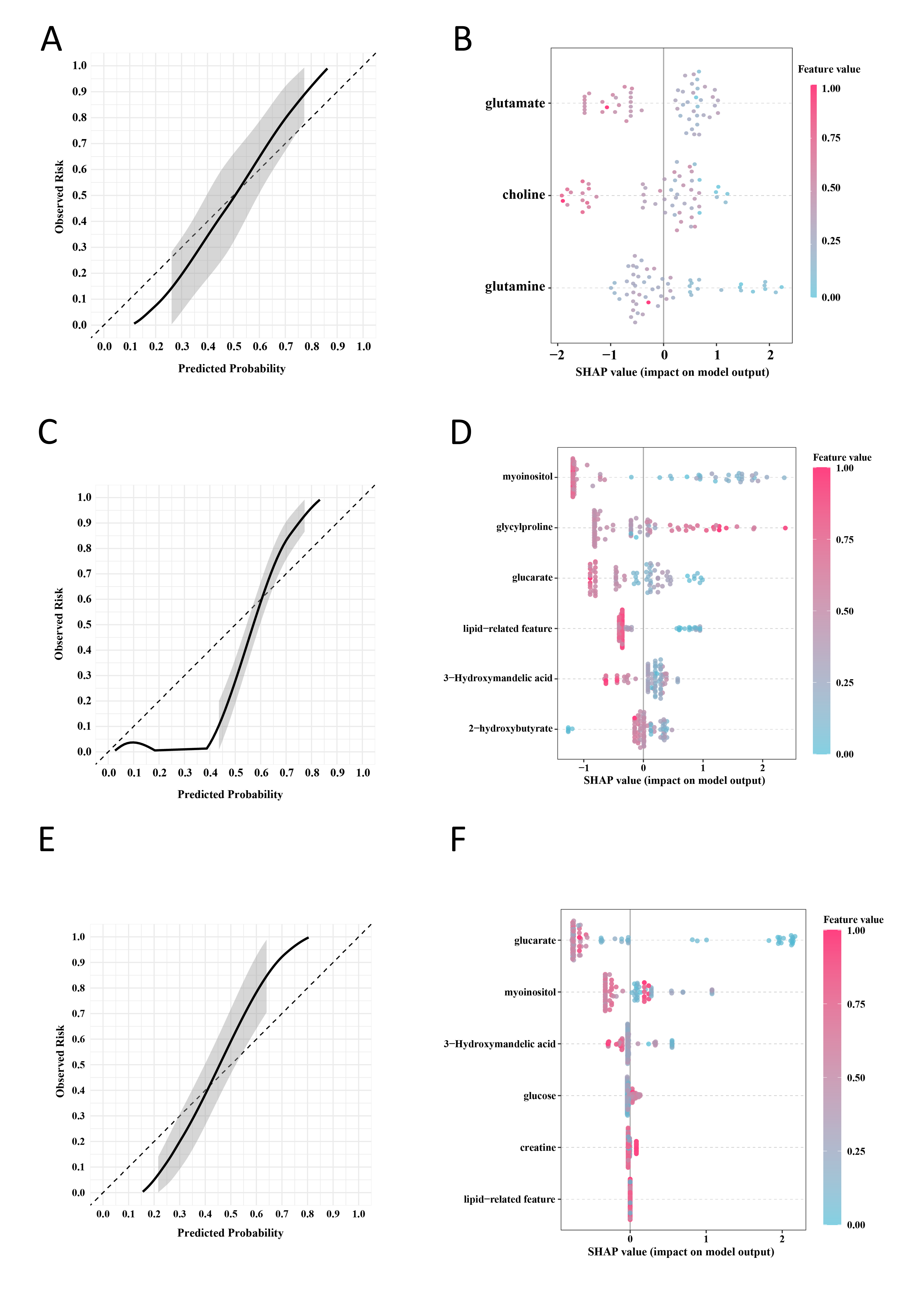

Supplement: S3 Fig — (C-D) (C) Calibration curves and (D) SHAP bee swarm plots for the PBID_PS and Control datasets. (E-F) (E) Calibration curves and (F) SHAP bee swarm plots for the VID_PS and Control datasets. (TIF) [file pone.0351295.s003.TIF]
